# Response to Damiani and colleagues

**DOI:** 10.1186/s13613-020-00757-6

**Published:** 2020-10-14

**Authors:** J-L Diehl, N. Peron, A. Philippe, D. M. Smadja

**Affiliations:** 1 Université de Paris, Innovative Therapies in Haemostasis, INSERM, 75006 Paris, France; 2 Biosurgical Research Lab (Carpentier Foundation), 20 rue Leblanc, AH-HP, Georges Pompidou European Hospital, 75015 Paris, France; 3 Medical Intensive Care Unit, AH-HP, Georges Pompidou European Hospital, 20 rue Leblanc, 75015 Paris, France; 4 Hematology Department, AH-HP, Georges Pompidou European Hospital, 75015 Paris, France

**To the Editor,**

We have read with great interest the comment of Damiani on our article, retaining the hypothesis of a possible major role of microvascular derangement in the physiopathology of COVID-19 ARDS. Such a hypothesis, supported by a number of arguments such as the rich expression of the SARS-CoV-2 ACE2 receptors in lung endothelial cells and dysregulation of the renin–angiotensin system, is now widely mentioned by others, using different approaches such as EIT studies [1], high-energy CT studies [2] and histopathology studies [3].

Damiani et al. have put our results in perspective with their own published observations of an inverse relationship between sublingual perfused vessel density and D-dimers in mechanically ventilated patients with severe SARS-CoV-2 pneumonia. They also reported a tendency to a decrease in sublingual microcirculation in patients with increased driving pressures. Interestingly, we observed in our 22 patients an inverse relationship between circulating endothelial cells (CECs) and total respiratory system compliance (*r* = − 0.278, *p* = 0.017), which could suggest a parallel between microvascular and alveolar insults, perhaps in relation with the hemodynamic consequences of a more severe alteration in respiratory mechanics. To explore if COVID-19 ARDS patients could exhibit a lung-specific microvascular response to high PEEP levels, as compared to non-COVID-19 ARDS patients, seems to be an important field of investigation.

One important point is that the very vast majority of studies in COVID-19 ARDS patients used, by convenience, ventilatory ratio (VR) as a marker of impaired ventilatory efficacy, as mentioned in Damiani's comment, rather than dead space measurements. However, it must be pointed out that VR was not originally designed to be used as a surrogate of dead space [4]. Accordingly, although highly significant, we found only a moderate level of correlation between VR and physiological dead space (*V*_*D*_/*V*_*T*_) [5]. The level was even lower than the values found in the non-COVID-19 ARDS literature.

We appreciate the opportunity to discuss some very important technical points in relation to capnography methods and the derived indexes. Damiani et al. state that our* V*_*D*_/*V*_*T*_ measurements could be inaccurate, due to transport delay of gas together with variable sampling flow rate. However, this point (and others also considered as disadvantages of the side stream method) is counterbalanced by specific disadvantages of the mainstream method which could also influence the precision of the results. Altogether, it is generally considered that there are advantages and disadvantages of both, the choice between them being of personal preference or from availability (as in our cohort of COVID-19 ARDS) rather than from strong recommendation [6]. Nevertheless, we agree that knowledge of physiologic and technologic basis of capnography is absolutely mandatory, both for research purposes and also for monitoring of ICU patients [7].

Finally, it will be important to further precisely investigate the relationship between dead space measurements, with a special focus on indicators of alveolar dead space, and markers of endothelial dysfunction, such as bio-markers (such as CECs and D-dimers) and innovative methods such as the video-microscopy methods used by Damiani and colleagues. Ideally, such prospective studies should include COVID-19 ARDS patients and non-COVID-19 ARDS patients as a control group. They should include measurements performed at different PEEP levels. They should also include measurements performed during the full course of invasive mechanical ventilation.

## Data Availability

The datasets used and/or analyzed during the current study are available from the corresponding author on reasonable request.
